# Induced Chirality and Vibrational Optical Activity in an Ionic‐Liquid Anion

**DOI:** 10.1002/anie.202502885

**Published:** 2025-05-30

**Authors:** Tom Frömbgen, Katrin Drysch, Thierry Tassaing, Thierry Buffeteau, Oldamur Hollóczki, Barbara Kirchner

**Affiliations:** ^1^ Mulliken Center for Theoretical Chemistry University of Bonn Beringstraße 4 53115 Bonn Germany; ^2^ Institut des Sciences Moléculaires (ISM), UMR 5255 University of Bordeaux – CNRS – Bordeaux INP Talence 33400 France; ^3^ Department of Physcial Chemistry University of Debrecen Egyetem tér 1 Debrecen 4032 Hungary

**Keywords:** Chirality, Computational chemistry, Ionic liquids, Molecular dynamics, VCD Spectroscopy

## Abstract

Here, we show that the four conformers of the regularly used ionic liquid anion bis(trifluoromethylsulfonyl)imide ${\left[{\mathrm{NTf}}_{2}\right]}^{-}$ are true enantiomeric pairs by analyzing their calculated vibrational circular dichroism spectra. The significant modes involve those atoms of the anion that form specific hydrogen bond patterns with the chiral probe molecule propylene oxide. Adding this probe molecule to the ionic liquid 1‐ethyl‐3‐methylimidazolium bis(trifluoromethylsulfonyl)imide, the experimental and simulated spectra indicate intermolecular interaction between the chiral molecule and the liquid and thus the induction of chirality in the anion. This emergence of chirality in the ionic liquid takes place via the hydrogen bonding between the anion of the ionic liquid and the solute, which results in a redistribution of the symmetric occurrence of conformers to an asymmetric one. Altogether, this study reveals the mechanism of chiral induction from the propylene oxide molecule to the ionic liquid anion, while pointing out the importance of rational over a random choice of the ionic liquid building units.

## Graphical abstract

Chirality can be induced in an ionic liquid anion. Normally, the enantiomeric conformers are equally populated, and no optical activity is observed. However, if a chiral probe molecule (e.g., propylene oxide) is added, a non‐symmetric redistribution of the conformers occurs, chirally imprinting the ionic liquid.

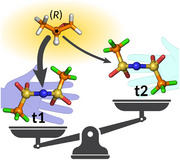

Chirality^[^ ^1^ ^]^ is a fundamental concept in chemistry,^[^ ^2^ ^]^ which often has tremendous effects on the chemical and biological activity of compounds. Since the importance of this structural feature had been recognized, significant advances have been made in controlling chirality in order to obtain enantiomerically pure substances. The underlying approaches rely on the difference between the two enantiomers in interacting with their environment and how chirality is transferred from one molecule to the other.

In general, chirality transfer^[^ ^3^ ^]^ refers to the process in which a chiral entity can turn a non‐chiral center or supra‐molecular material^[^ ^4^, ^5^ ^]^ into permanently chiral structures.^[^ ^6^, ^7^, ^8^ ^]^ In contrast, chiral induction, directed from a chiral solute to a non‐chiral solvent, can be considered an expansion of the solute's asymmetry into its solvent shell through triggering a temporal shift in the distribution of the adjacent solvent molecules' conformations, making it apparently chiral. Thus, these interactions amplify local chirality, and a chiral medium emerges through a chiral imprint, which has the potential to improve discrimination between chiral compounds in the system. This effect is non‐permanent, as the solvent molecule is again free to occupy all conformations after being exchanged by another in the solvent shell. In this paper, we will use the term “chiral induction” for such non‐permanent solvent effects. However, it is important to stress here that just before permanent chirality emerges in chirality transfer through, e.g., the formation of a covalent bond, the reacting molecules must be correctly aligned to each other, which must be the consequence of effects identical to those in chiral induction. In this sense, chiral induction is the first step of chirality transfer as well, which makes it an elusive, yet highly influential effect.^[^ ^9^, ^10^ ^]^

Ionic liquids (ILs) have been used as reaction media for decades, often resulting in more efficient processes than in common organic molecular solvents. They were, for example, employed in catalysis,^[^ ^11^, ^12^, ^13^, ^14^, ^15^ ^]^ ${\mathrm{CO}}_{2}$ capture,^[^ ^16^, ^17^, ^18^, ^19^ ^]^ and other organic reactions^[^ ^20^, ^21^, ^22^ ^]^ but also in inorganic synthesis.^[^ ^23^, ^24^, ^25^, ^26^ ^]^ The explored reactions include a number of asymmetric reactions as well, using chiral catalysts.^[^ ^22^, ^27^, ^28^, ^29^ ^]^ However, for the present discussion, it is worth mentioning the efforts made in using chiral ILs as solvents for synthesis to introduce the desired asymmetry into the product through the reaction medium itself.^[^ ^28^, ^30^, ^31^, ^32^ ^]^ For the success of this approach, it is imperative to understand the process of transferring chiral information between the IL solvent and the solute.

Examples of the above‐described chirality induction in ILs have been investigated through theoretical and experimental techniques.^[^ ^27^, ^33^, ^34^, ^35^, ^36^, ^37^, ^38^ ^]^ Oulevey et al. discovered that an enantiomerically pure amino acid anion induces chirality in the 1‐ethyl‐3‐methyl‐imidazolium cation, [C2C1Im]+.^[^ ^34^ ^]^ They pointed out the importance of selecting the right ion for the IL medium when designing asymmetric or stereoselective syntheses in these solvents.^[^ ^34^ ^]^ The thereby emerging asymmetry has been identified in vibrational circular dichroism (VCD) spectra as well. VCD spectroscopy is a powerful tool to trace such chirality transfer/induction effects and determine the absolute configuration of molecules and materials.^[^ ^39^, ^40^, ^41^, ^42^, ^43^ ^]^ Our group investigated the effect of a chiral probe molecule on the optical activity of a solution of chiral butan‐2‐ol in 1‐ethyl‐3‐methylimidazolium (*S*)‐alaninate.^[^ ^37^ ^]^ We found that the interactions with the achiral cation are decisive in discriminating between the (*R*) and (*S*) stereoisomers of the solute.

Thus, the hitherto explored chiral induction effects were restricted to the IL cation. However, in many cases, the anion is dominant in the interactions of the IL with a variety of solutes, including water, ${\mathrm{CO}}_{2}$,^[^ ^44^, ^45^, ^46^, ^47^, ^48^ ^]^ and other molecules with cationic or hydrogen bonding sites.^[^ ^49^, ^50^, ^51^ ^]^ Thus, it is reasonable to assume that chiral induction by the solute would also occur in the anion, and hence this hypothesis is necessary to consider when such a solution is to be understood. In a previous, purely theoretical work, we suggested that an induction to the anion of an IL is possible.^[^ ^38^ ^]^

In this paper, we demonstrate through theoretical chemical methods and experimental VCD spectra how the chirality of a propylene oxide can induce optical activity in one of the most often used IL anions,^[^ ^52^, ^53^, ^54^ ^]^ bis(trifluoromethylsulfonyl)imide, ${\left[{\mathrm{NTf}}_{2}\right]}^{-}$, in an IL medium. With a good agreement between the simulated and measured spectra, our results indicate that chiral induction amplifies the asymmetry of the mixture through a so far unexplored manner, opening novel channels to design more efficient procedures toward enantiomerically pure substances.

Defined by its two C–S–N–S dihedrals, the ${\left[{\mathrm{NTf}}_{2}\right]}^{-}$ anion has altogether four conformations, two *trans* (t1 and t2) and two *cis* (c1 and c2),^[^ ^38^, ^54^, ^55^ ^]^ as shown in Figure 1. These structures can be found as minima on the potential energy surface by static quantum chemical calculations, and can also be observed as distinct peaks in the combined distribution function of the two corresponding dihedrals from trajectories of ab initio molecular dynamics (AIMD) simulations of the bulk IL (Figure 2 left). The difference between the conformers in their structure and symmetry is apparent, making the pairs c1 and c2 as well as t1 and t2 true enantiomers of each other.

**Figure 1:**
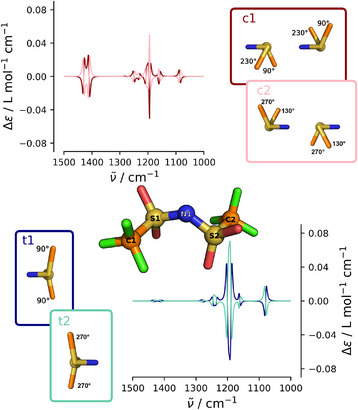
Schematic illustration of relevant conformers of the ${\left[{\mathrm{NTf}}_{2}\right]}^{-}$ anion and their VCD spectra. Center: Ball‐and‐stick representation of ${\left[{\mathrm{NTf}}_{2}\right]}^{-}$ with relevant atoms labeled explicitly. The color code of the atoms is C: orange, N: dark blue, O: red, F: light green, S: gold, with O and F atoms omitted for clarity. Top right and bottom left (in colored boxes): Newman‐like projections of the anion conformers c1 and c2 ($C_{1}$ symmetry, top), as well as t1 and t2 ($C_{2}$ symmetry, bottom), showing the molecule as it is viewed from along the S–S axis. Conformers are defined through the characteristic dihedral angles ∠(C1‐S1‐N1‐S2) and ∠(C2‐S2‐N1‐S1) (see Ref. [55]), given in black for each conformer. Top left and bottom right: VCD spectra of the conformers (top left and bottom right) were obtained through static calculations (c1: maroon; c2: pink, t1: blue; t2: green). Please note that the VCD spectra are scaled by a factor of 1.085 for consistency with other theoretical spectra presented in this manuscript.

**Figure 2:**
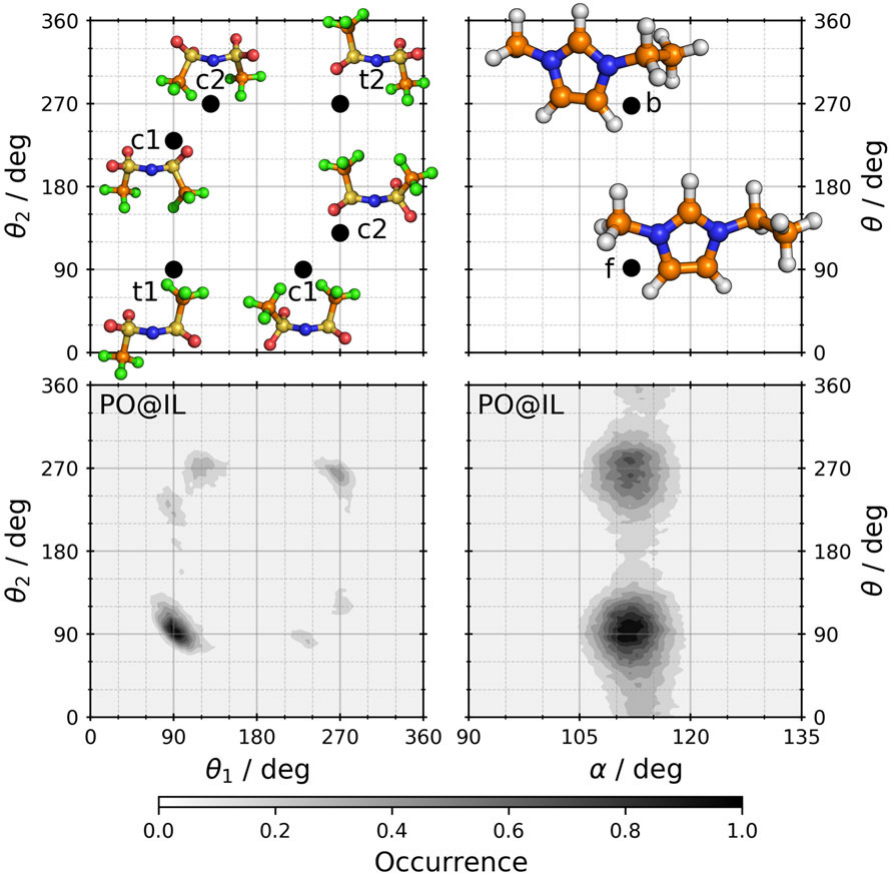
Conformational distribution plots for ${\left[{\mathrm{NTf}}_{2}\right]}^{-}$ (left column panels) and [C2C1Im]+ (right column panels) from AIMD simulations. In the top row panels, locations of the conformers (minima on the potential energy surface) are illustrated by black dots and corresponding labels for guidance. The anion (left) has four conformers, c1, c2, t1, and t2, defined by the dihedrals $\theta_{1}=∠$(C1‐S1‐N1‐S2) and $\theta_{2}=∠$(C2‐S2‐N1‐S1), see Figure 1 for their structure and labels. The cation (right) has two conformers, b (back) and f (front), that indicate the orientation of the ethyl side chain and are defined by the angle α=∠(N1‐C2‐C3) and dihedral θ=∠(C1‐N1‐C2‐C3), see Figure S10 for atom labels. Bottom row panels show the results obtained from the simulation with (*R*)‐propylene oxide dissolved in the IL.

To track these conformations spectroscopically, VCD spectroscopy is the ideal tool,^[^ ^3^, ^56^, ^57^, ^58^, ^59^, ^60^, ^61^, ^62^ ^]^ since the ${\left[{\mathrm{NTf}}_{2}\right]}^{-}$ anion has multiple IR active modes. The VCD spectra predicted by static quantum chemical calculations for each conformer (depicted in Figure 1) support this idea, showing varying positive and negative peaks at the respective vibrational frequencies, which have opposite signs for the corresponding enantiomers.

Our calculated IR spectra of the anion's conformers align well‐besides the obvious and well‐known solvent effects and shifts due to computational methods—with the experiment (see Figure S1) and their main features arise from the following modes: We observe asymmetric S–O in‐phase and out‐of‐phase vibrational modes at around $1400\;cm^{-1}$. The symmetric S–O in‐phase and out‐of‐phase modes can be found at 1140 ${\mathrm{cm}}^{-1}$. The band at around $1060\;cm^{-1}$ is assigned to the asymmetric stretching of the SNS group, while the vibrational modes related to the asymmetric stretching of the ${\mathrm{CF}}_{3}$ groups can be observed at about 1200 and 1230 ${\mathrm{cm}}^{-1}$. A detailed literature discussion and assignment of these vibrational normal modes can be found in Sections S1.1 and S1.2.

The pure IL does not show any optical activity, due to the dynamic and symmetric equilibrium between the (chiral) conformers that cancels out these effects. However, according to the spectra in Figure 1, if this equilibrium is asymmetrically distorted, the system is expected to show an overall vibrational optical activity that could be traced through VCD spectroscopy. In the present case, we suspect that the interaction of the solvent molecules—here the ions — with chiral substances (propylene oxide) should influence the population distribution of their conformers. To verify this hypothesis, we performed AIMD simulations on an IL solution of a single (*R*)‐propylene oxide (PO). We chose [C2C1Im][NTf2] as a solvent with simulation details given in section S2 of the Supporting Information. The solvation of PO in this IL is key to understanding the principles that govern the conformational landscape of the IL anions in the vicinity of the solute.

Each peak in the combined distribution function of the two dihedrals (Figure 2 left) can be attributed to one of the conformations, and therefore their integral quantifies their occurrence. The distribution is highly asymmetric for the *trans* conformers, and yields overall populations of 50.8%, 15.6%, 13.7%, and 19.8% for conformers t1, t2, c1, and c2, respectively (see Section S2.7 of the Supporting Information for details on the integration scheme). There is also a slight asymmetry in the structure of the cations as well (Figure 2 right), which has been discussed previously in extended detail^[^ ^37^ ^]^ and additional analyses are presented in Section S3.3 of the Supporting Information. It is noteworthy that the existing standard force field‐based simulations are not capable of reproducing this chiral induction (see also Sections S3.1, S3.2 of the Supporting Information). If in the future such simulations are desired, either polarizable force fields or specially fitted force fields need to be applied.^[^ ^63^ ^]^

Since the chiral carbon atom of PO and the hydrogen attached to it bear a positive partial charge, it seems reasonable to assume that the IL anions are located at this optically active site. This chiral site should, therefore, force a certain orientation and conformation onto a nearby anion, and thereby transferring chiral information to it. In Figure 3, we clearly observe the close proximity of the fluor and oxygen atoms of the anion to the hydrogen atom at $C^{★}$ chiral center of PO. These distances are around 200pm, thus we can infer that the chiral induction manifests via this kind of $C^{★}$–H⋯O/F hydrogen bond. For the hydrogen bond accepted by the oxygen atom, the linear arrangement is clearly highly occupied, see dark spots at $\alpha =180^{\circ }$ for the oxygen atom plot right in Figure 3. In contrast, the hydrogen bond accepted by the fluorine atom exhibits greater variability in the adopted angles ranging from 180 to around 90.

**Figure 3:**
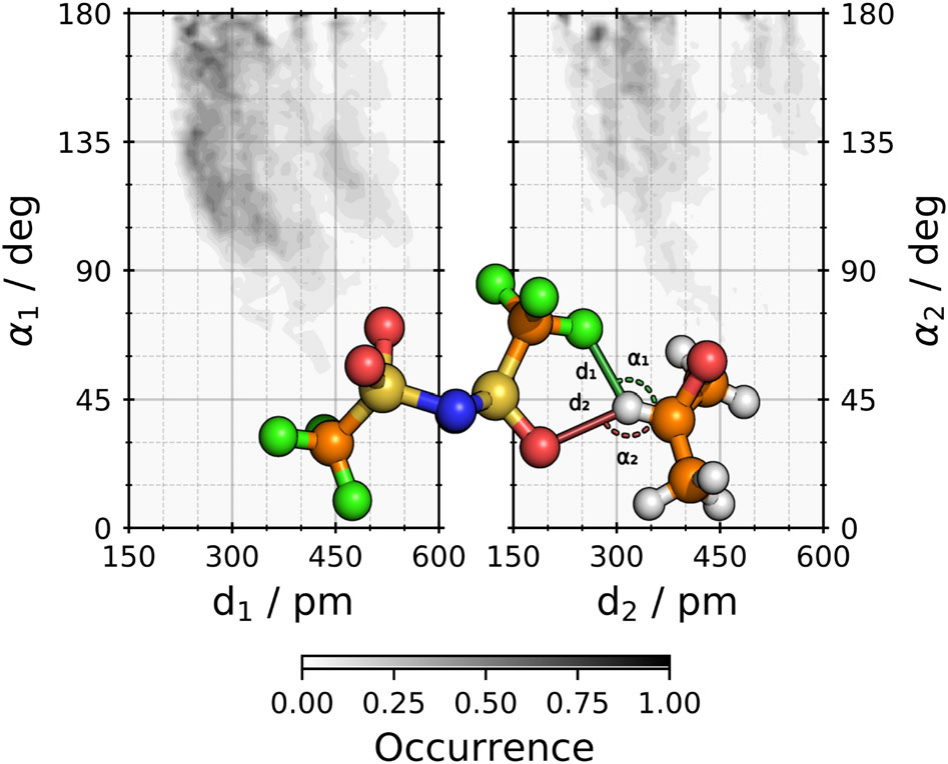
Structural arrangement of ${\left[{\mathrm{NTf}}_{2}\right]}^{-}$ and PO found in the AIMD simulation. Center: Ball‐and‐strick representation of an ${\left[{\mathrm{NTf}}_{2}\right]}^{-}$–PO dimer with relevant geometric quantities highlighted. The color code of the atoms is H: light grey, C: orange, N: dark blue, O: red, F: light green, S: gold. Distances between the H atom attached to the chiral $C^{★}$ atom of PO to the F/O atom of ${\left[{\mathrm{NTf}}_{2}\right]}^{-}$ are labeled as $d_{1}$/$d_{2}$, respectively. The $C^{★}$–H–F/O angles are referred to as $\alpha_{1}$/$\alpha_{2}$, respectively. Left and right panels show combined distribution functions, correlating the distribution functions of $d_{1}$ and $\alpha_{1}$ (left) as well as those of $d_{2}$ and $\alpha_{2}$ (right). Please note that a nearest neighbor criterion with respect to $d_{1}$ and $d_{2}$ was applied.

Turning finally to the VCD spectra and in agreement with the discussion above, we observe that the PO@IL system exhibits a very prominent VCD spectrum when predicted from AIMD simulations (Figure 4 second thin red spectrum from above). The dominant (+/−/+/−/+) pattern observed between 1100 and $1300\;cm^{-1}$ can be assigned to the asymmetric stretching of the ${\mathrm{CF}}_{3}$ groups. Likewise, the trisignate (−/+/−) pattern observed around $1400\;cm^{-1}$, corresponding to the asymmetric stretching of the ${\mathrm{SO}}_{2}$ groups, and the negative peak at $1050\;cm^{-1}$, corresponding to the asymmetric stretching of the SNS group. The advantage of the theoretical approach lies in the possibility to dissect the spectrum into individual contributions of the various species, pinpointing the physical effects behind the optical activity of the solution. In accordance with the assignment of the peaks, plotting the spectrum of the anions (Figure 4 thick red spectrum from above) separately produces an almost identical band structure to that of the full spectrum, showing that the optical activity of the solution can be almost completely attributed to the ${\left[{\mathrm{NTf}}_{2}\right]}^{-}$. In blue, we show a spectrum that uses the isolated ions calculated by static methods in the same percentage as obtained from AIMD simulations; however, the features cannot satisfactorily be reproduced by the weighted averages spectrum.

**Figure 4:**
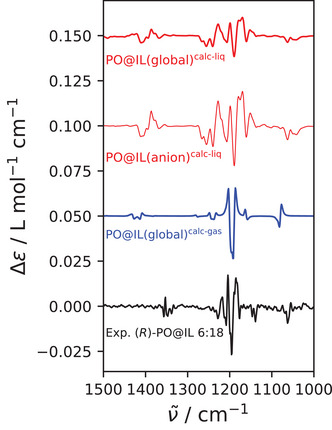
Theoretical and experimental VCD spectra of (*R*)‐PO@IL. The two red lines correspond to bulk phase spectra calculated from the AIMD simulation. The topmost spectrum is a global spectrum, i.e., it includes all molecules present in the AIMD simulation, whereas the spectrum below is obtained by only including the anions. The blue line represents the VCD spectrum that is obtained as weighted averages of the (static) individual spectra of the four anion conformers (c1, c2, t1, t2) and the two cation conformers (b, f). Black line represents the corrected experimental VCD spectrum ((*R*)‐PO@IL – (*S*)‐PO@IL)/2. Please note that the theoretical VCD spectra are scaled by a factor of 1.085 to better align with the experimental spectrum.

To put these exciting results onto solid ground, we also provide an experimental VCD spectrum in black in Figure 4. It shows a striking resemblance to the simulated ones, verifying our calculations, despite the notable broadening of the central band structure around $1200\;cm^{-1}$ in the theoretical spectra. This resemblance even includes the split peak on the right‐hand side of the dominant (+/−/+/−/+) pattern.

Our theoretical and experimental results show evidence that chirality is induced in the anion of an IL solvent by a chiral solute, creating a chiral environment. This phenomenon is highly important for using these liquids as solvents in asymmetric or stereoselective synthesis, since choosing the right IL for the given purpose may apparently involve tuning the anion to create the ideal chiral environment within the liquid for maximizing selectivity.

## Supporting Information

In the attached Supporting Information, we provide details on: experimental setup and the measurements of IR and VCD spectra; computational details regarding the calculations and simulations performed, including the employed force fields, and discussion of the methodology used to determine the conformer abundance; additional results including anion conformer populations in other simulations, structural arrangement of the cation, and information on the calculation of vibrational spectra. 

## Conflict of Interests

The authors declare no conflict of interest.

## Supporting information

Supporting Information

## Data Availability

The data that support the findings of this study are available in the Supporting Information of this article.
