# Supplementary material for: Induced Chirality and Vibrational Optical Activity in an Ionic‐Liquid Anion
Source: Angew Chem Int Ed Engl. 2025 May 30;64(28):e202502885. doi: 10.1002/anie.202502885 (PMC12232900; doi:10.1002/anie.202502885)
Supplement: Supplementary file 1 — Supporting Information [file ANIE-64-e202502885-s001.pdf]

Supporting Information: Induced Chirality and Vibrational Optical Activity in an Ionic-Liquid Anion

Tom Frömbgen,[†,§] Katrin Drysch,[†,§] Thierry Tassaing,[*,‡] Thierry Buffeteau,[*,‡] Oladmur Hollóczki,[*,¶] and Barbara Kirchner[*,†]

†*Mulliken Center for Theoretical Chemistry, University of Bonn, Beringstraße 4, 53115 Bonn, Germany*

‡*Institut des Sciences Moléculaires (ISM), UMR 5255, University of Bordeaux – CNRS – Bordeaux INP, 33400 Talence, France*

¶*Department of Physcial Chemistry, University of Debrecen, Egyetem tér 1, 4032 Debrecen, Hungary*

§*T.F. and K.D. contributed equally to this work.*

E-mail: thierry.tassaing@u-bordeaux.fr; thierry.buffeteau@u-bordeaux.fr; holloczki.oldamur@science.unideb.hu; kirchner@thch.uni-bonn.de

1 Experimental Data

1.1 Experimental setup

VCD measurements of (R)-PO@IL and (S)-PO@IL samples were performed using a ThermoNicolet Nexus 670 FTIR spectrometer equipped with a VCD optical bench.[S1] In this optical bench, the light beam was focused on the sample by a BaF_2 lens (191 mm focal length), passing an optical filter, a BaF_2 wire grid polarizer (Specac), and a ZnSe photoelastic modulator (Hinds Instruments, Type II/ZS50). The light was then focused by a ZnSe lens (38.1 mm focal length) onto a $1 \times 1 mm^2$ HgCdTe (ThermoNicolet, MCTA* E6032) detector. (R)-PO@IL and (S)-PO@IL mixtures of 2 PO for 3 IL were prepared by adding 15 mg of (R)-PO or (S)-PO into 152 mg of $[C_2C_1Im][NTf_2]$. Then, 2 µL of this mixture was dropped onto a BaF_2 window and pressed with a second BaF_2 window in order to have a film thickness around 2.8 µm.

IR absorption spectra of (R)-PO@IL and (S)-PO@IL samples were recorded after the sample preparation, and once every day during the VCD experiment. We emphasize that the VCD spectra are continuously measured and results from the average of block of spectra saved every 1 hour. Since the two windows are not sealed and PO is very volatile, the concentration of PO decreases with time and becomes close to zero after 4 days (see fig. S1, right panel). VCD spectra of (R)-PO@IL and (S)-PO@IL samples were recorded during the first 4 days corresponding to an average concentration of 1 PO for 3 IL, and during 4 days after to record the VCD spectra of IL alone. These last VCD spectra were subtracted to the VCD spectra of (R)-PO@IL and (S)-PO@IL samples in order to eliminate the VCD artifacts coming from the strong absorption of the $[NTf_2]^-$ anion (see Supporting Information, Figure S2). Finally, since the two VCD spectra of (R)-PO@IL and (S)-PO@IL samples are not perfect mirror images, the half difference between the two VCD spectra has been calculated to eliminate VCD artifacts arising from our optical setup.

All spectra were recorded at a resolution of $4 cm^{-1}$. The photoelastic modulator was

adjusted for a maximum efficiency in the mid-IR region at 1400 cm^{-1}. Calculations were performed via the standard ThermoNicolet software, using Happ and Genzel apodization, de-Haseth phase-correction and a zero-filling factor of one.

1.2 IR spectra of (*R*)-PO/IL mixtures

The experimental IR spectrum of (*R*)-PO@IL sample is reported in fig. S1 (left panel) in the 1000 cm^{-1} to 1500 cm^{-1} spectral range. The most intense bands in the IR absorption spectrum are due to the $[NTf_2]^-$ anion and appear below 1400 cm^{-1}. Their assignment has been reported in the literature.[S2,S3] The bands at 1352 and 1332 cm^{-1} are attributed to the in-phase and out-of-phase asymmetric stretching of the SO_2 groups, respectively. The intensity ratio of these two bands gives information about the conformation (trans or cis) of the $[NTf_2]^-$ anion. The band at 1140 cm^{-1} gathers the in-phase and out-of-phase symmetric stretching of the SO_2 groups. The band at 1058 cm^{-1} is assigned to the asymmetric stretching of the SNS group. Finally, both the most intense band around 1195 cm^{-1} and the shoulder at 1228 cm^{-1} are related to the asymmetric stretching of the CF_3 groups. The bands related to the $[C_2C_1Im]^+$ cation are observed with very weak intensity at wavenumbers above 1400 cm^{-1}, as shown in fig. S1 (right panel). The bands at 1431 cm^{-1} and 1471 cm^{-1} are attributed to CH bending modes coupled with CN stretching mode, whereas the broad band around 1456 cm^{-1} is related to bending of CH_2 group and asymmetric bending of CH_3 groups.[S4] Finally, in this region only the band at 1408 cm^{-1} is related to the propylene oxide species. This band is attributed to the symmetric bending of CH_3 group. As shown in fig. S1 (right panel), the intensity of the 1408 cm^{-1} band decreases with time, and its intensity is close to zero after 4 days, since this compound is very volatile.

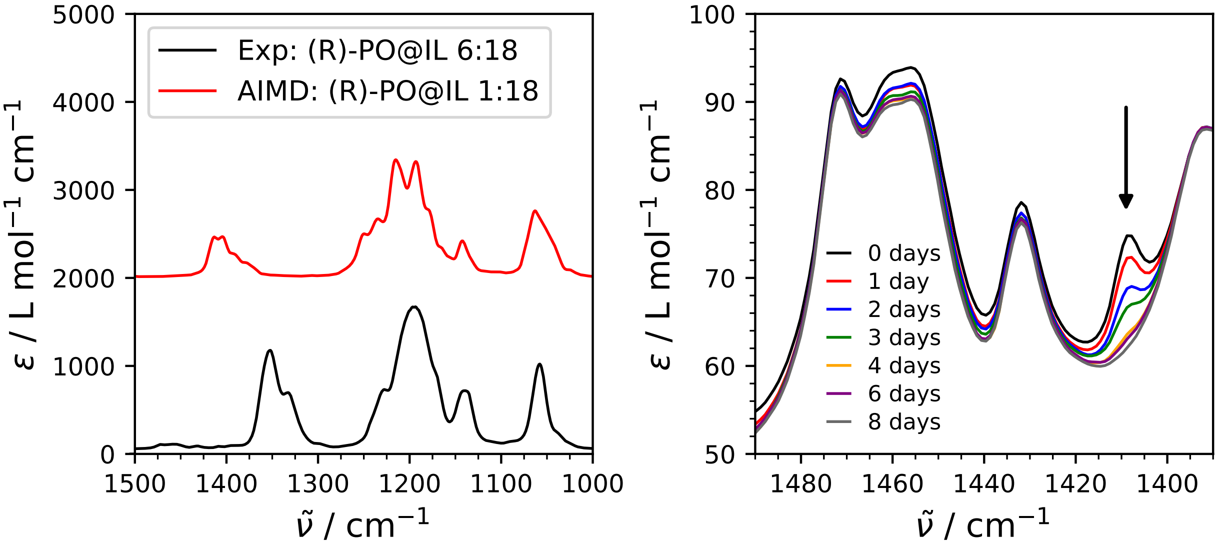

Figure S1: Left: Experimental (black) and calculated (red) IR spectra of (R)-PO@IL. The calculated IR spectrum was scaled by a factor of 1.085. Right: Experimental IR spectra of (R)-PO@IL as a function over a period of days. The arrow points to the contribution of PO that decreases over time while the component evaporates.

1.3 VCD spectra of (*R*)- and (*S*)-PO@IL mixtures

The raw VCD spectra of (R)-PO@IL and (S)-PO@IL samples are reported in fig. S2 (left panel) in the 1000 cm^{-1} to 1500 cm^{-1} spectral range. These spectra reveal VCD signatures for the strong absorption bands of the $[NTf_2]^-$ anion. Opposite contributions are observed for the two enantiomers of propylene oxide, suggesting a chiral induction of the chiral molecule to the bis(trifluoromethylsulfonyl)imide anion. Since the two VCD spectra are not perfectly image mirror, the half difference between the two VCD spectra has been calculated to eliminate VCD artifacts from our optical setup. The corrected VCD spectrum of (R)-PO/IL sample is presented in the main text (Figure 4) and is compared to the AIMD (R)-PO/IL simulation.

In the right panel of fig. S2, the corresponding g factors are presented ($\Delta\varepsilon/\varepsilon$), where ε is the corresponding IR spectrum, presented in fig. S1 (left panel, black line). For the asymmetric stretching mode of the CF_3 groups, the g factor does not exceed $2 \cdot 10^{-5}$.

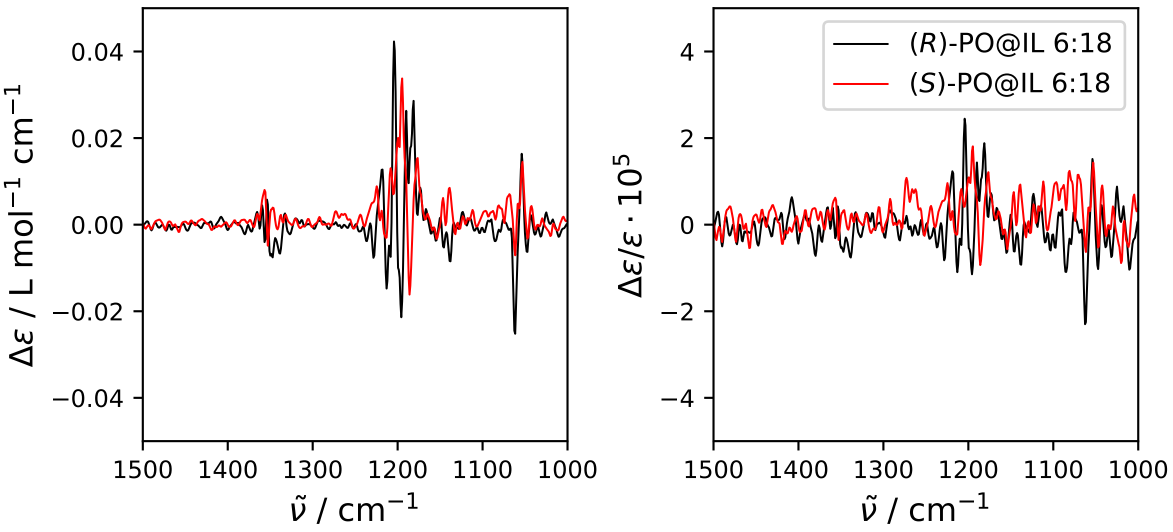

Figure S2: Left: Differential raw spectra between (R)-PO@IL or (S)-PO@IL mixtures and the raw VCD spectrum of the pure IL. The experimental conditions were: 7.5 mg of PO in 152 mg of $[C_2C_1Im][NTf_2]$, 2.8 µm path length between 2 BaF_2 windows, 4 days acquisition time, 4 cm^{-1} resolution. Right: Experimental g factor ($\Delta\varepsilon/\varepsilon$) of ($R$)-PO@IL and ($S$)-PO@IL spectra. In order to increase the signal to noise ratio of the $\Delta\varepsilon/\varepsilon$ spectra outside the absorption bands, an offset of 200 was added to the ε spectra.

2 Theoretical methodology

2.1 Systems investigated

In the course of this study, various molecular dynamics simulations, including force field based nonpolarizable, polarizable and *ab initio* simulations, were performed. For all setups of force field based simulations, several replica simulations were carried out, as recommended for example by Coveney and coworkers,[S5] Zuckerman and coworkers[S6] and in a recent *JCIM* editorial.[S7] However, no replica simulations for AIMD setup were performed, due to their very high computational costs.
For all simulations, cubic boxes were used. The density for the *ab initio* and the nonpolarizable system including PO were obtained by a weighted average of the liquid phase densities of the IL[S8] and PO,[S9] assuming an ideal mixing behavior.

An overview of the different simulation setups is presented in table S1.

Table S1: Overview of the systems simulated in this study: System name, number of ion pairs N_{IP} of the ionic liquid $[C_2C_1Im][NTf_2]$, number of (R)-propylene oxide molecules N_{PO}, number of replica simulations n_{rep}, the equilibration and production run times t_{equil}, t_{prod} (in ps), as well as the temperature of the production run T_{prod} (in K), density ρ (in $g\,cm^{-3}$) and box size a (in Å) of the simulations for each system.

System	N_{IP}	N_{PO}	n_{rep}	t_{equil}	t_{prod}	T_{prod}	ρ	a	Ref.
				ab initio					
$PO@IL^{revPBE}_{298K}$	18	1	1	50	30	298	1.478	19.9822	current
$PO@IL^{revPBE}_{350K}$	18	1	1	15	30	350	1.478	19.9822	S10
$PO@IL\text{-}2^{revPBE}_{350K}$	18	1	1	45	30	350	1.478	19.9822	current
$PO@IL^{SCAN}_{350K}$	18	1	1	15	30	350	1.478	19.9822	current
				polarizable					
$IL^{CL\&Pol}_{350K}$	18	0	20	2000	2000	350	1.514	19.7683	current
$PO@IL^{CL\&Pol}_{350K}$	18	1	20	2000	2000	350	1.478	19.9822	current
				nonpolarizable					
$IL^{CL\&P}_{350K}$	18	0	20	2000	2000	350	1.514	19.7683	current
$PO@IL^{CL\&P}_{350K}$	18	1	20	2000	2000	350	1.478	19.9822	current
IL^{NGOLP}_{350K}	18	0	20	2000	2000	350	1.514	19.7683	current
$PO@IL^{NGOLP}_{350K}$	18	1	20	2000	2000	350	1.478	19.9822	current

2.2 Quantum chemical calculations

2.2.1 Static VCD spectra calculations

Quantum chemical calculations using density functional theory (DFT) were conducted for the different conformations of the $[C_2C_1Im]^+$ cation and the $[NTf_2]^-$ anion, separately, in order to produce reference vibrational circular dichroism (VCD) spectra that serve as a point of comparison with the results obtained from AIMD simulations. All calculations were performed using the Turbomole 7.8 program package.[S11–S13] The BP86[S14,S15] generalized gradient approximation (GGA) functional was employed with the def2-TZVP[S16] basis set. The D3(BJ)[S17,S18] dispersion correction scheme was employed to account for dispersion effects. Molecular geometries were optimized at the aforementioned level of theory, and subsequent frequency calculations were performed to generate the normal modes, from which the VCD

spectra were obtained. The calculations were conducted utilizing a $10^{-8}\,E_\mathrm{h}$ convergence criterion for the SCF energy and a maximum norm of Cartesian gradient up to $10^{-3}\,a_0$. The spectra were modeled with a Lorentzian band shape and a full width at half maximum of $8\,\mathrm{cm}^{-1}$.

2.2.2 Conversion of VCD rotation strength into VCD intensity

The VCD rotational strength (R) was obtained from the static VCD spectra calculations described above in units of $10^{-44}\,\mathrm{esu}^2\,\mathrm{cm}^2$. In order to compare these with the VCD intensity ($\Delta\epsilon$) of the experimental spectrum, which is given in $\mathrm{L\,cm^{-1}\,mol^{-1}}$, the VCD rotational strength is converted the following way:[S19]

$$\Delta\varepsilon = \frac{\nu}{2.296\cdot 10^{-3}} \cdot \left(\frac{\mathrm{d}R}{\mathrm{d}\nu}\right) \quad . \tag{1}$$

2.3 AIMD Simulations

AIMD simulations (refer to table S1) were performed using the mixed Gaussian and plane wave approach as implemented in the Quickstep module of CP2k.[S20,S21] The procedure for conducting the AIMD simulation for the $\mathrm{PO@IL}^{\mathrm{revPBE}}_{\mathrm{350K}}$ system is outlined below:

1. Construction of a cubic simulation box, containing 18 IP of $[C_2C_1Im][NTf_2]$ and 1 molecule of propylene oxide, using PACKMOL.[S22]

2. Classical MD simulation at 350 K using LAMMPS,[S23] serving as a pre-equilibration.

3. AIMD simulation in the canonical ensemble (constant number of particles, volume and temperature), with a timestep of 0.5 fs using CP2k.[S20] Interatomic forces were calculated by means of DFT, applying the revPBE[S24] functional (GGA) and the Goedecker–Teter–Hutter pseudopotentials for the core electrons.[S25–S27] Dispersion corrections were included using the DFT-D3(BJ) method,[S17,S18] and the double-zeta basis set MOLOPT-DZVP-SR-GTH was used for all atoms.[S28]

(a) 10 ps equilibration at elevated temperature (400 K) with massive thermostats to activate all molecular vibrations.

(b) 5 ps relaxation to the target temperature (350 K) using a standard global thermostat.

(c) 30 ps production at 350 K. The coordinates were dumped in every step, i.e., each trajectory contains 60 000 frames.

(d) Calculation of the vibrational spectra based on our previous works.[S29,S30]

A similar procedure was employed for the simulation of the IL system, with the key difference being that the simulation box was constructed containing only 18 ion pairs of $[C_2C_1Im][NTf_2]$. For the PO@IL-2^{revPBE}_{350K} system, the simulation of the PO@IL was extended under the same conditions for another 30 ps. The simulation of the PO@IL^{revPBE}_{298K} system was conducted by equilibrating the final step of the PO@IL^{revPBE}_{350K} simulation for 5 ps at the target temperature of 298 K, followed by a 30 ps production run at the same temperature. For the PO@IL^{SCAN}_{350K} simulation, the same procedure as described for the PO@IL^{revPBE}_{350K} was followed, with the only differences being the application of the SCAN[S31] functional (meta-GGA) instead of the revPBE functional and the usage of corresponding GTH-SCAN pseudopotentials.[S25]

2.4 Force-Field based MD Simulations

The nonpolarizable MD simulations (refer to table S1) were carried out using LAMMPS.[S23] For the NGOLP systems, the $[C_2C_1Im]^+$ cation was modeled using the CL&P[S32–S37] force field, while $[NTf_2]^-$ was modeled by the (Neumann, Golub, Odebrecht, Ludwig, Paschek) NGOLP[S38] force field. For the CL&P and CL&Pol systems, the IL (i.e., both cation and anion) was modeled using the CL&P[S32–S37] and CL&Pol[S39,S40] force fields for nonpolarizable and polarizable simulations, respectively. Propylene oxide was modeled by the OPLS-AA[S41] force field (see section 2.5). Within these force fields, polarizability for heavy atoms (except for hydrogen) is modeled using the classical Drude oscillator model.[S42] The Drude particles

(DPs) were assigned masses $m_{\mathrm{DP}} = 0.4\,\mathrm{g\,mol^{-1}}$ and charges $q_{\mathrm{DP}} = \sqrt{\alpha k}$, where α is the polarizability of the atom the DP is attached to and $k_{\mathrm{DP}} = 4184\,\mathrm{kJ\,mol^{-1}\,\AA^{-2}}$ is the force constant of the DP vibration.

The simulation inputs were generated using fftool[S43] and PACKMOL.[S22] All simulations obeyed the following protocol. For each system, five independent simulations were performed with identical initial configurations but different seeds for velocity initialization.

1. Construction of a cubic simulation box containing the molecules outlined in table S1, using fftool[S43] and PACKMOL.[S22]

2. Creating the input files for a nonpolarizable system using fftool.[S43]

3. For the polarizable systems:

   (a) Addition of Drude induced dipoles to the LAMMPS[S23] data file.

   (b) Scaling of the Lennard-Jones parameters ε between fragments to prevent double counting of induction effects and σ to adjust the density.

4. Carrying out the simulations in the NVT ensemble with a timestep of 1.0 fs using LAMMPS.[S23]

   (a) 2 ns of equilibration at 350 K. A temperature-grouped dual Nosé–Hoover thermostat[S44] was employed for the polarizable simulations, with the Drude cores and nonpolarizable atoms residing at the target temperature, while the Drude particles were kept at 1 K. For the nonpolarizable MD simulations, the Nosé–Hoover thermostat was employed.[S45,S46] We note that the total energies of all systems are well-converged within this time scale.

   (b) 2 ns production at 350 K, using the aforementioned thermostat settings. The coordinates were dumped at a frequency of 1000 steps, i.e., each trajectory contains 2000 frames.

2.5 Force field parameters for propylene oxide

The force field parameters to model propylene oxide were taken from the OPLS All-Atom Force Field[S41] and are listed in table S2. The equilibrium distances between the PO molecule and $[NTF_2]^-$, $[C_2C_1Im]^+$ or another PO molecule (required for the simulations with polarizable force fields) were obtained from ORCA[S47] calculations on a R2SCAN-3c[S48] level of theory and are displayed in table S3.

Table S2: Assignment of OPLS-AA atom types to the atoms of the PO molecule. For atom labels, see fig. S3.

atom	C1	C2	O3	H4	H5	C6	H7	H8	H9	H10
OPLS-AA	C3O	C3O	OY	HC	HC	CT	HC	HC	HC	HC

Table S3: Equilibrium distances $R_{\mathbf{eq}}$ (in Å) between the centers of masses of the PO-PO, PO-$[NTF_2]^-$ and PO-$[C_2C_1Im]^+$ dimer. The distances are required to scale the Lennard-Jones parameters in the CL&Pol force field. Results were obtained on an R2SCAN-3c level of theory.

system	distance
PO-PO	3.918
PO-$[NTf_2]^-$	4.576
PO-$[C_2C_1Im]^+$	4.633

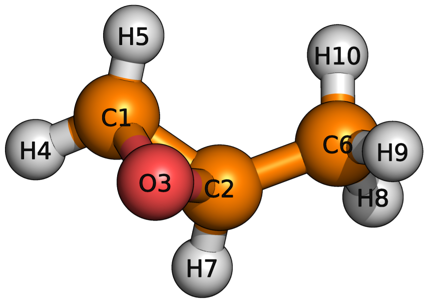

Figure S3: Ball-and-stick structure of propylene oxide, including atomic labels for comparison in table S2.

2.6 Data analysis and visualization

All trajectory analyses (CDFs, vibrational spectra) were carried out with TRAVIS.[S29,S30,S49–S51] The calculated vibrational spectra from AIMD simulations were scaled by a factor of 1.085 for comparison to the experiment. Molecular images in ball-and-stick representation were created in PyMol[S52] and VMD.[S53] All other figures were created using matplotlib.[S54]

2.7 Integration methodology for determining relative populations of $[NTf_2]^-$ conformers

To analyze the conformational landscape of the $[NTf_2]^-$ anion, we base our evaluations on the approach proposed by Canongia Lopes et al.[S55] In their study, specific configurations of the two proper C−S−N−S dihedral angles determine whether a trans or cis conformer is present. When both dihedral angles adopt 90° or 270°, a trans conformer is observed. Conversely, dihedral angle pairs (90°, 130°) or (270°, 230°) result in a cis conformer. Four unique conformers can be identified, as visualized in Fig. 1 of the main text, while their locations in the two-dimensional combined distribution function (CDF) is shown in fig. S4. We note here that the conformers t1 and t2, as well as c1 and c2 are enantiomers of each other.

To determine the relative populations of the conformers (i.e., their abundance in the simulations), the population of each angle combination for the two C−S−N−S dihedral angles is tracked throughout the production run of the simulations and stored as a CDF, a two-dimensional matrix. These populations are normalized by dividing each value by the maximum observed population. When multiple replica simulations are conducted, the CDFs calculated from all trajectories are summed before normalization. To quantify the relative populations of the individual conformers, a clustering approach is employed. This method considers both the derivatives in x and y directions, as well as the normalized population for each angle combination in the CDF. An angle combination is considered significant if either

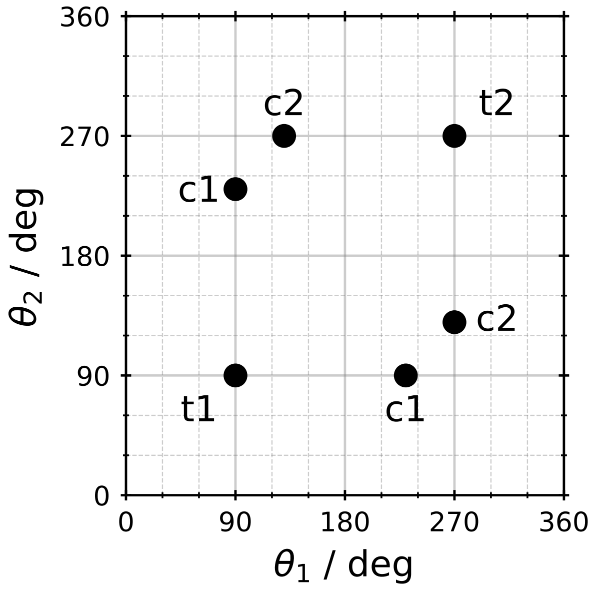

Figure S4: Location of the different conformers c1, c2, t1 and t2.

of the following criteria is met.

1. The magnitude of the gradient $|\vec{v}|$ of the normalized population P in the x- and y-directions exceeds a defined cutoff a: $|\vec{v}| = \sqrt{\left(\frac{\mathrm{d}P}{\mathrm{d}x}\right)^2 + \left(\frac{\mathrm{d}P}{\mathrm{d}y}\right)^2} > a$.

2. The normalized population P of that angle combination exceeds a defined cutoff b: $P > b$.

Once all significant data points are identified, contiguous regions are determined. Each region is associated with the conformer located within that region (see fig. S4). Additionally, fig. S5 displays two exemplary CDFs, with significant angle combinations indicated by colored arrows pointing in the direction of the gradient vector, $\vec{v} = \frac{\mathrm{d}P}{\mathrm{d}x} \cdot \vec{e}_x + \frac{\mathrm{d}P}{\mathrm{d}y} \cdot \vec{e}_y$ where $\vec{e}_x$ and $\vec{e}_y$ are the unit vectors in x- and y-directions respectively. The arrows are colored to indicate the conformer they are assigned to. To determine the relative populations of each conformer, the normalized populations of all significant angle combinations assigned to that conformer are summed and then divided by the total sum of all normalized populations. By choosing the cutoffs a and b it is important to consider as much of the population as possible by

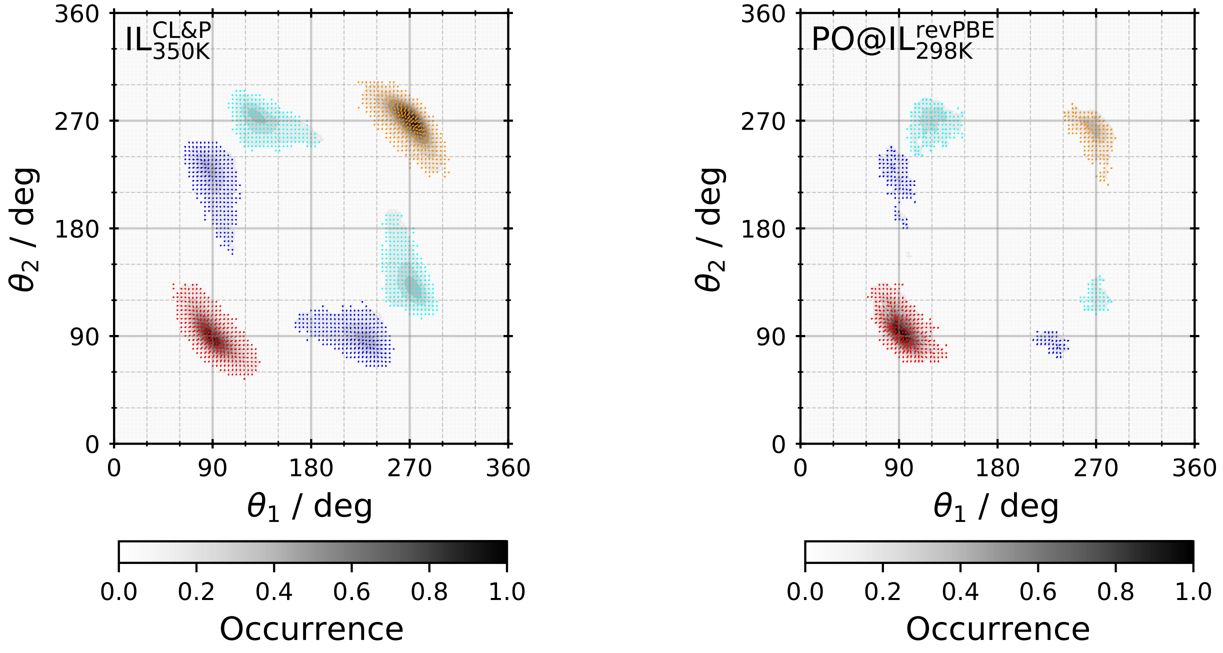

Figure S5: CDFs of $IL^{CL\&P}_{350K}$ and $PO@IL^{revPBE}_{298K}$, with significant angle combinations indicated by colored arrows, assigning each to its corresponding conformer. The color code is as follows: red: t1, blue: c1, cyan: c2, orange: t2.

avoiding the merging of distinct areas. For this application, values between 0.03 and 0.05 for a and values between 0.10 and 0.13 for b have been found to be optimal.

We also explore a second approach, purely based on geometric criteria, for integrating the CDFs. To calculate the populations of different conformers, the CDF matrices are divided as illustrated in fig. S6. As for the cluster integration approach, the populations are normalized by dividing each value by the maximum observed population. When multiple replica simulations are conducted, the populations for each dihedral angle pair are summed before normalization. The normalized populations within the integration areas are summed up and divided by the total normalized population to determine the relative abundance of each conformer.

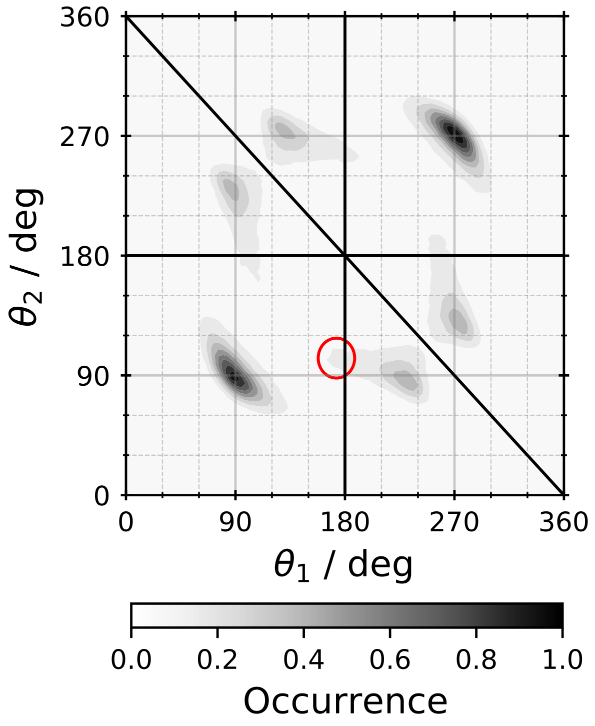

Figure S6: Partitioning of the conformational space of $[NTf_2]^-$ using geometric criteria, indicated by black lines, visualized for an example system. A potential problem of the geometric approach is highlighted in red.

A challenge of the first approach is to choose the cutoffs a and b meaningfully to keep a balance between including highly populated regions of the conformational space (colored spots in fig. S5) and neglecting (chemically irrelevant) sparsely populated regions (gray areas). At the same time, the cutoffs must prevent distinct regions from merging, as this would hinder the ability to subdivide the populations. On the other hand, given carefully chosen constraints, the first approach offers a chemically motivated selection of integration areas. The second approach circumvents this issue by integrating the entire CDF and strictly assigning population to a certain conformer. However, this approach has a limitation: in some cases, regions that are extensions of one of the cis conformers are mistakenly assigned to one of the trans conformers. This issue is highlighted in fig. S6, where the affected area is marked by a red circle.

To ensure a chemically meaningful assignment of populations to conformers, we decided

to follow the first approach (cluster integration) in this study.

3 Additional results

3.1 Interconversion barriers among the $[NTf_2]^-$ conformers

The interconversion barriers among the anion conformers as well as their relative stabilities were calculated using ORCA 5.0.4[S47] at the B3LYP-D3(BJ)/def2-TZVPP[S56–S60] level of theory and are visualized in Figure S7. The barrier for the direct conversion between t1 to t2 has been estimated in Refs.[S55] and[S61] to be significantly higher than the conversion between t1/c1 or t2/c2 and c1/c2, hence the rearrangements between the conformers should occur via the pathways presented here.

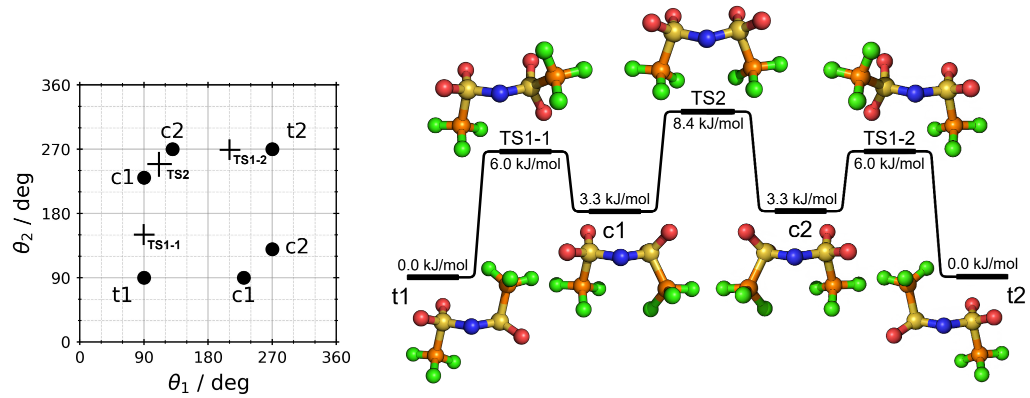

Figure S7: Visualization of the interconversion barriers among the conformers as well as their relative stabilities.

3.2 Anion conformer populations observed in other AIMD simulations

In fig. S8, additional combined distribution functions (CDFs) are provided, displaying the prevalent conformations of the anion, $[NTf_2]^-$, observed in different AIMD simulations. We note that the data of the top left panel is already presented in the bottom left panel of Figure 2 of the main text, and given here just for comparison. The top right panel features data from a previous work.[S10] In the bottom left panels, data from additional simulations

of PO@IL at 350 K are presented. It is important to note that while the simulations show some differences in the population of conformers (see table S4), a strong preference for the t1 conformer is observed in all systems. This is additional evidence for the observed chirality induction effect.

In particular, as shown in fig. S9, the t1 conformer is strongly favored in close vicinity to PO. This lets us conclude that PO induces the t1 conformation in $[NTf_2]^-$.

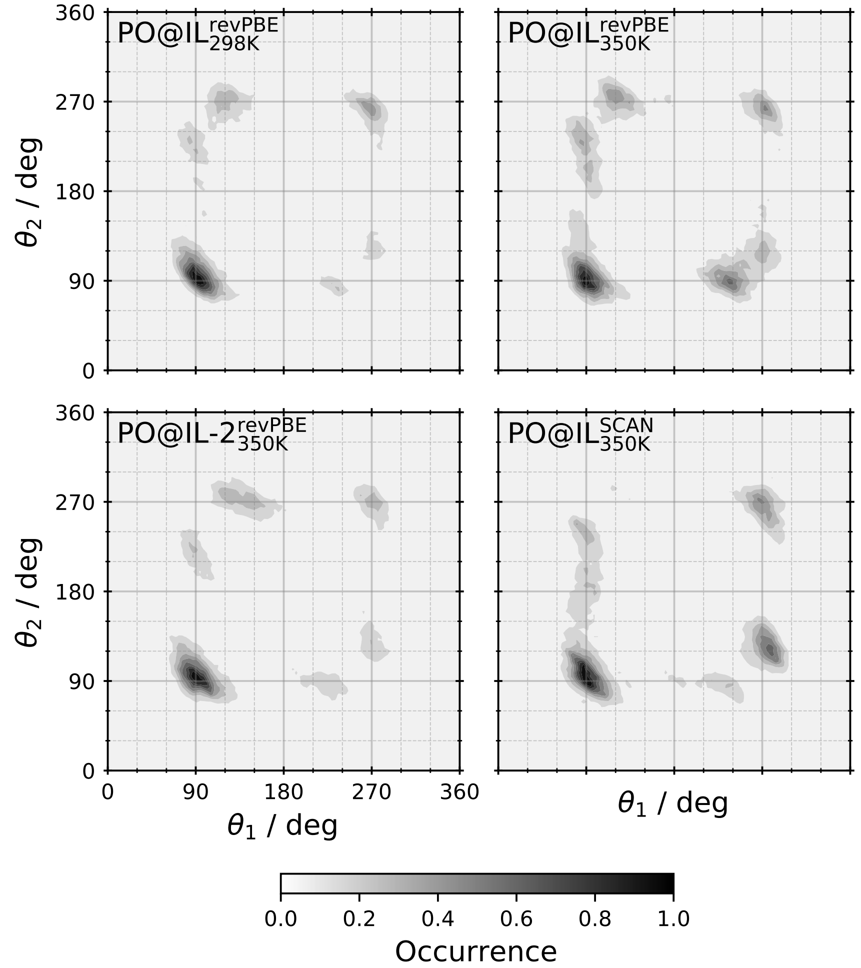

Figure S8: Conformational distribution plots for $[NTf_2]^-$ conformers calculated from *ab initio* molecular dynamics simulations. The system label (see table S1 for details) is given in the top left corner of each panel. The occurrence was normalized to unity. See fig. 1 of the main text for definitions of θ_1 and θ_2.

Table S4: Relative abundance (in %) of the four conformers of $[NTf_2]^-$ in the AIMD systems defined in table S1. The relative abundance is determined from a cluster integration of the corresponding CDFs.

System	t1	t2	c1	c2
$PO@IL^{revPBE}_{298K}$	50.8	15.6	13.7	19.8
$PO@IL^{revPBE}_{350K}$	36.0	10.1	41.1	12.8
$PO@IL\text{-}2^{revPBE}_{350K}$	49.9	9.8	15.5	24.8
$PO@IL^{SCAN}_{350K}$	39.7	16.4	21.9	22.0

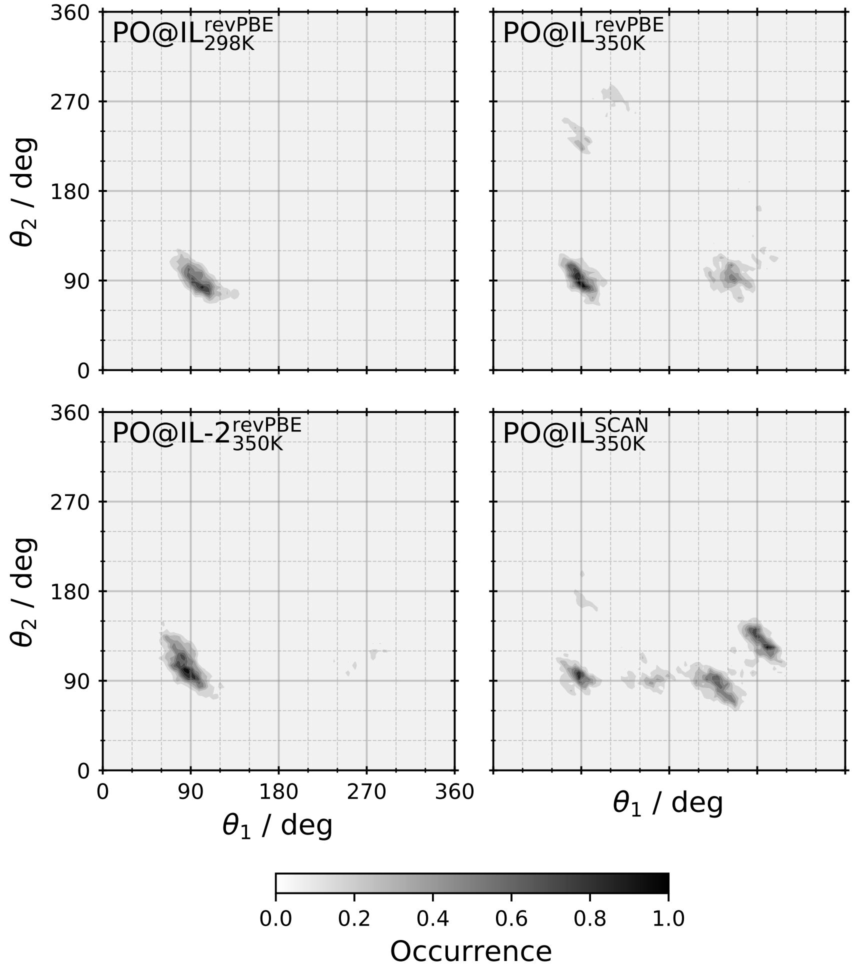

Figure S9: Conformational distribution plots for $[NTf_2]^-$ conformers calculated from *ab initio* molecular dynamics simulations with the next neighbor criterion towards the propylene oxide applied. The system label (see table S1 for details) is given in the top left corner of each panel. The occurrence was normalized to unity. See fig. 1 of the main text for definitions of θ_1 and θ_2.

3.3 Anion conformer populations observed in force field based MD simulations

The conformational distribution plots for the $[NTf_2]^-$ conformers based on the simulations of the CL&P, NGOLP and CL&Pol systems are displayed in fig. S10. The corresponding relative abundance and standard deviation of the four conformers is given in table S5.

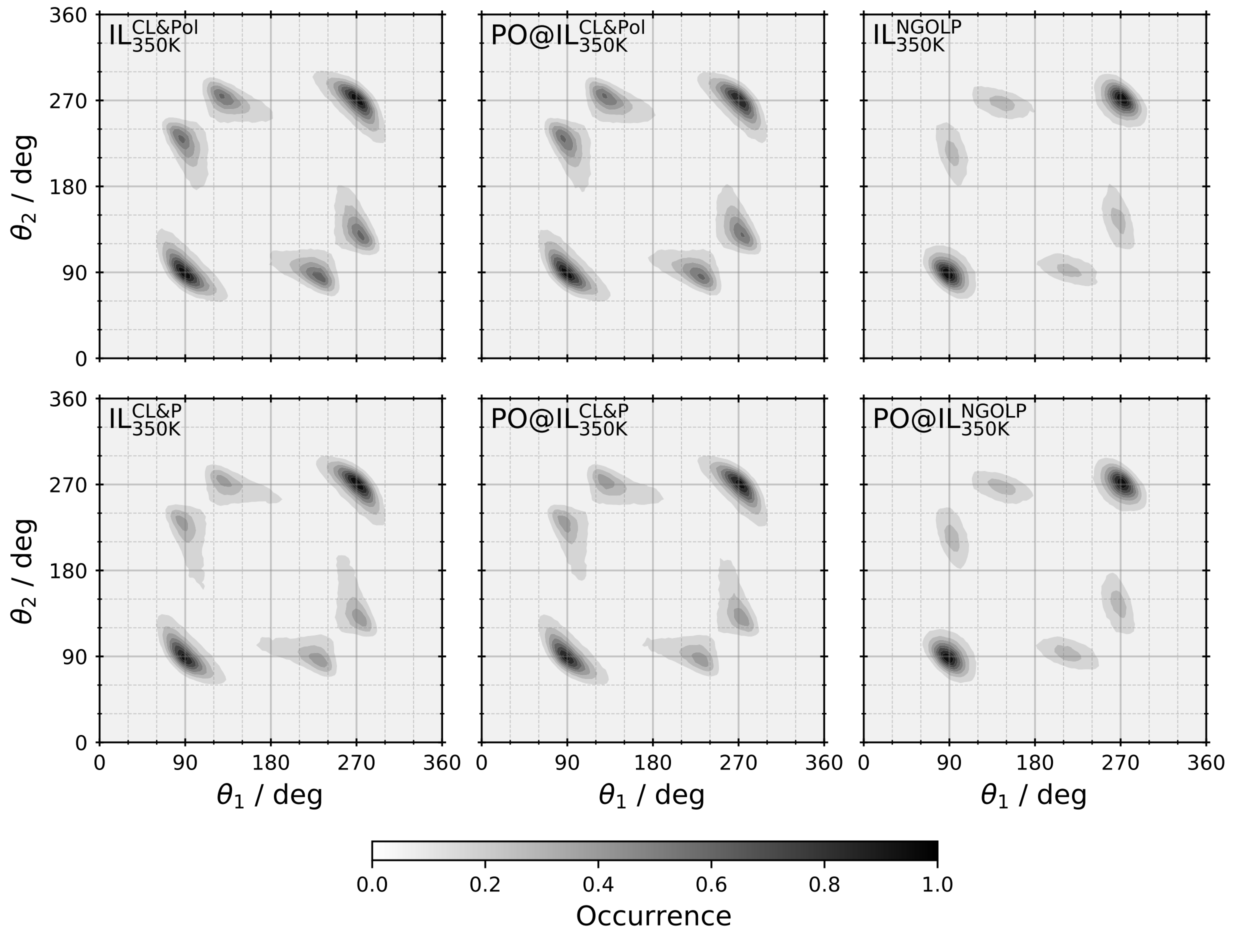

Figure S10: Conformational distribution plots for $[NTf_2]^-$ conformers calculated from polarizable and nonpolarizable molecular dynamics simulations. The system label (see table S1 for details) is given in the top left corner of each panel. The occurrence was normalized to unity, and first averaged over all frames of the production run of a single trajectory, then averaged over all replica trajectories. See Fig. 1 of the main text for definitions of θ_1 and θ_2.

The provided relative abundance and standard deviation were derived through a bootstrapping procedure. For each system, 100 groups consisting of 20 CDFs were formed,

whereby CDFs can occur multiple times in a group. The mean values for these groups were calculated, and from these results, the overall mean values and standard deviation for each system were determined.

Table S5: Relative abundance and standard deviation (in %) of the four conformers of $[NTf_2]^-$ in the CL&P, NGOLP and CL&Pol systems defined in table S1. The relative abundance and standard deviation are determined as the mean value and standard deviation from cluster integrations of 100 groups containing 20 CDFs each.

System	t1	t2	c1	c2
$IL_{350K}^{CL\&Pol}$	26.3 ± 1.9	27.2 ± 1.9	23.6 ± 2.3	22.9 ± 1.8
$PO@IL_{350K}^{CL\&Pol}$	26.2 ± 1.7	27.1 ± 2.0	23.7 ± 2.0	23.0 ± 1.9
$IL_{350K}^{CL\&P}$	26.1 ± 1.7	27.3 ± 1.9	23.8 ± 2.1	22.8 ± 1.8
$PO@IL_{350K}^{CL\&P}$	26.2 ± 1.7	27.1 ± 1.8	23.8 ± 2.1	22.9 ± 1.6
IL_{350K}^{NGOLP}	26.3 ± 1.8	27.7 ± 1.8	23.4 ± 2.1	22.6 ± 1.6
$PO@IL_{350K}^{NGOLP}$	25.8 ± 1.6	27.3 ± 1.9	23.9 ± 1.9	23.0 ± 1.7

Similar results are observed for all systems, inasmuch as (i) the conformer distributions are very symmetric with respect to the populations of t1 vs. t2 as well as c1 vs. c2, and (ii) no significant change in the conformer distribution is observed when adding the chiral PO molecule. Thus, none of the force fields in this study, whether nonpolarizable or polarizable, was able to replicate the shift in the conformational distribution of the $[NTf_2]^-$ anion induced by the chiral PO molecule, as observed in the AIMD simulations.

3.4 Spatial alignment of the cation around propylene oxide

The cation, $[C_2C_1Im]^+$ is a rather rigid molecule and its only conformers are generated through the ethyl group rotation (i.e., technically, they are rotamers). The ethyl group rotation has three minima on the potential energy surface, but one minimum is high in energy and features a low barrier, which is why there are effectively two conformers populated in the simulations. These conformers are enantiomers of each other. For one enantiomer, the C1-

N1-C2-C3 dihedral angle, shown in fig. S11, measures about 90° and is referred to as "front" subsequently. For the other enantiomer, the angle is about 270°, and this configuration is termed "back" in the following.

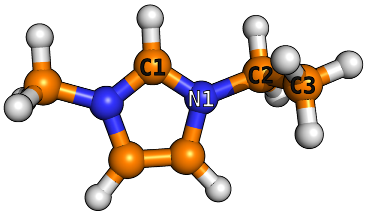

Figure S11: Ball-and-stick representation of the 1-ethyl-3-methyl-imidazolium cation, with a C1-N1-C2-C3 dihedral angle of approximately 90°. Color code: hydrogen: light grey, carbon: orange, nitrogen: dark blue.

Figure S12, left panel, shows the dihedral distribution function (DDF) for the C1-N1-C2-C3 angle in the 1-ethyl-3-methyl-imidazolium cation, obtained from the $\mathrm{PO@IL}_{298\mathrm{K}}^{\mathrm{revPBE}}$ simulation. The DDF including all cations is shown in black and reveals that overall, the front conformer is slightly more populated. Additionally, in red, the DDF of cations that match a nearest neighbor criterion to propylene oxide is shown. In close vicinity to propylene oxide, the back conformer is populated, exclusively.

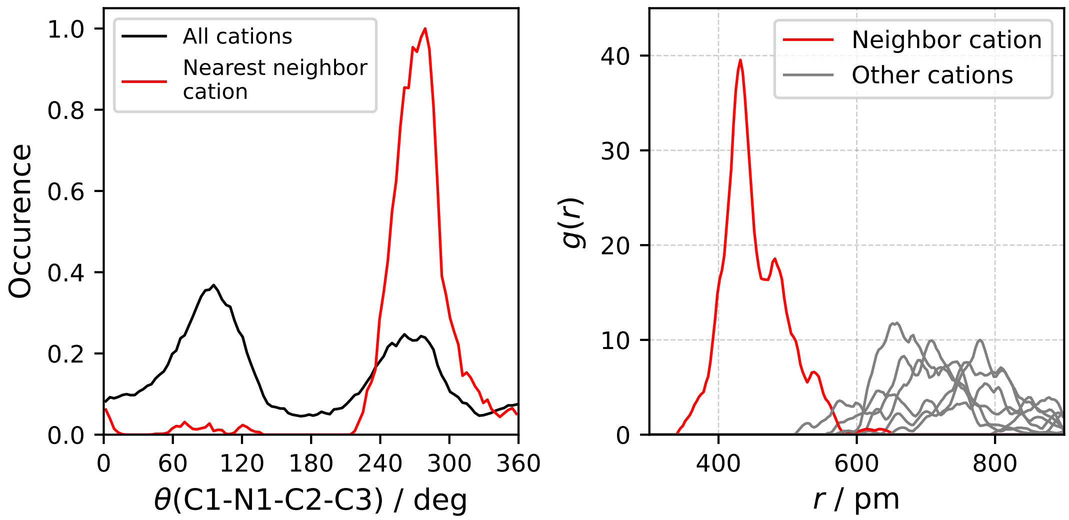

Figure S12: **Left:** Dihedral distribution function (DDF) for the C1-N1-C2-C3 angle in the 1-ethyl-3-methyl-imidazolium cation. The black line represents the DDF for all cations in the simulation, while the red line shows the DDF for which a nearest neighbor criterion of the cation (center of ring) towards the propylene oxide (center of mass) is met. **Right:** Radial distribution functions of cation (center of ring) and propylene oxide (center of mass) for all cations individual in the system. Cation meeting the next neighbor criterion are displayed in red, all others in grey.

To determine how many cations actually contribute to the DDF nearest neighbor condition are applied. For each cation in the system a radial distribution functions (RDF) between the cation (center of ring) and propylene oxide (center of mass) were calculated for each cation, individually, and are displayed in fig. S12, right panel. We find that the red peak in the DDF is originated by one particular cation (red RDF), while all other cations reside at larger distances with respect to propylene oxide (grey RDFs).

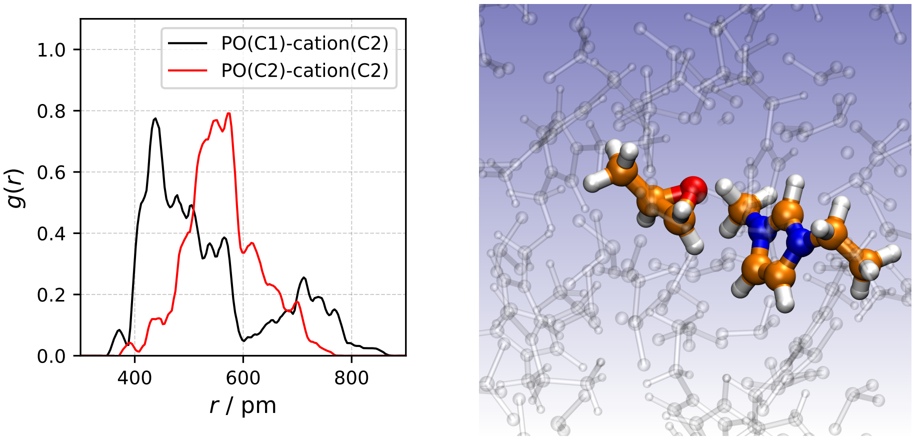

Figure S13: **Left:** RDFs of the C2 carbon atom of the nearest neighbor cation identified in fig. S12 and the C1 and C2 carbon atoms of propylene oxide. The data were smoothed using a Savitzky-Golay filter due to high noise. **Right:** Snapshot from the trajectory, showing the alignment of propylene oxide and the 1-ethyl-3-methyl-imidazolium cation.

Cation- propylene oxide RDFs are shown in fig. S13, left panel. The center of ring of the cation coordinates to the O3 and the H7 of the propylene oxide (see fig. S3). RDFs of the C2 carbon atom of the nearest neighbor cation identified in fig. S12 and the C1 and C2 carbon atoms of propylene oxide show from which direction the cation approaches the propylene oxide. Since the C2 carbon of the cation approaches the C1 carbon of the propylene oxide more closely than its C2 carbon, it can be inferred that the molecules orient themselves such that their sterically hindering groups are directed away from each other. A snapshot illustrating the spatial arrangement of the cation next to propylene oxide is displayed in fig. S13, right panel.

3.5 Technical setting of calculating VCD spectra in TRAVIS

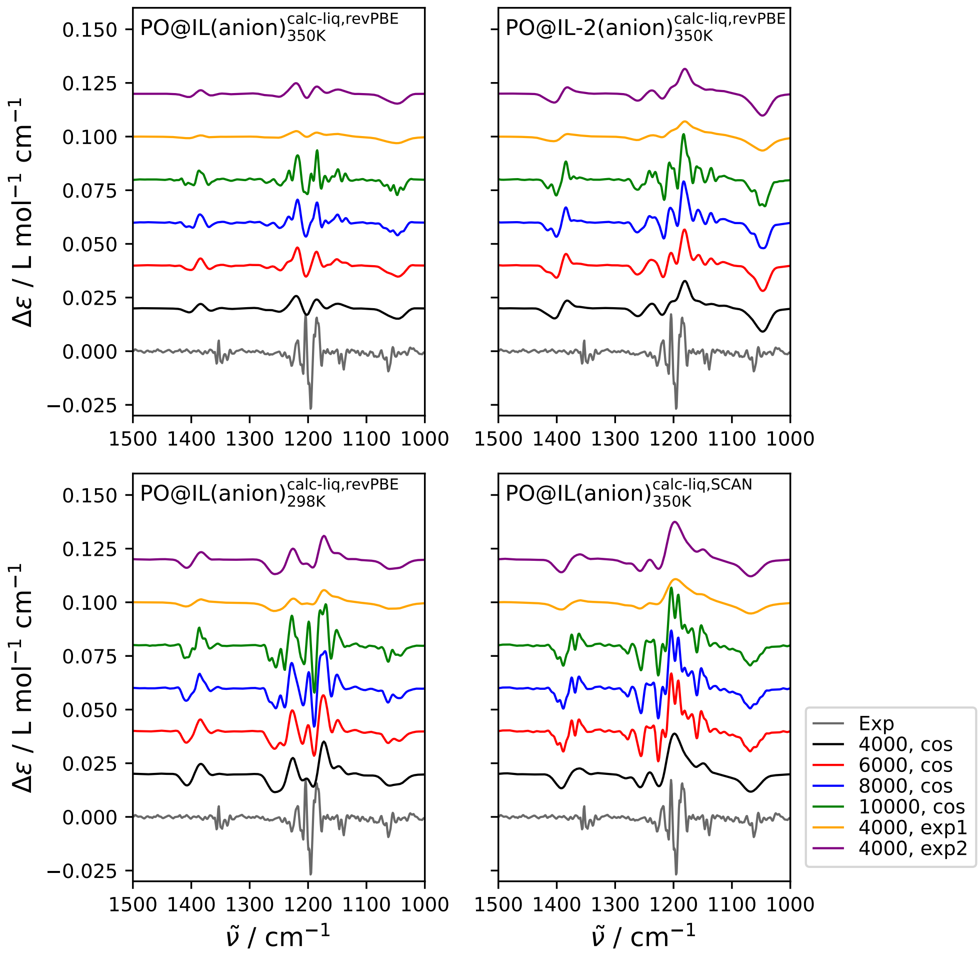

Figure S14: Different correlation depth for the VCD spectra generation based on the AIMD simulations investigated in this study. Please note that the spectra from simulations using the revPBE functional (top panels and bottom left panel) were scaled by 1.085 to match the experimental VCD spectrum (gray spectrum in the bottom of each panel), while the the spectra calculated from simulations using the SCAN functional (bottom right panel) were scaled by 1.04.

The theoretical IR and VCD spectra from AIMD simulations presented in this work are calculated using TRAVIS.[S49,S50] Detailed explanations on the methodology can be found in Refs. S29,S30,S51,S62,S63. It is important to note, that the spectra are calculated based on correlation functions of the molecular electric and magnetic dipole moments and a Fourier transform thereof. Various technical settings can be fine-tuned during the calculation of

the vibrational spectra in TRAVIS. The influence of the most important settings, namely correlation depth and window function, are illustrated in fig. S14.

A window function is usually multiplied with the correlation function to reduce undesired side lobes of the peaks that stem from the discrete and finite nature of the correlation function. Several types of window functions are known in the literature, such as cosine, exponential or Gaussian type functions.

The correlation depth describes how many trajectory frames are used for the correlation with any other trajectory frame. A small correlation depth will result in very broad peaks and low resolution, while a large correlation depth gives rise to a higher resolution and sharp peaks at the cost of more noise. Thus, a compromise in terms of resolution and noise has to be found.

As a default, TRAVIS uses a correlation depth of 4000 frames, and applies the Hann function, a cosine type function as a window function, which is an overall good choice. In the present case, however, we found that a cosine function in combination with a correlation depth of 10000 frames (green lines in fig. S14) to be more suited to illustrate the induced chirality observed in our simulations.

3.6 Theoretical global VCD spectra

In fig. S15 we present the VCD spectra resulting from the contributions of the propylene oxide and all ions of the ionic liquid for all systems. As outlined in the main paper, by comparing the global VCD spectra to the VCD spectra obtained only from the $[NTf_2]^-$ anion contributions one notices, that the global spectra of each simulation are always governed by the spectra of the anions.

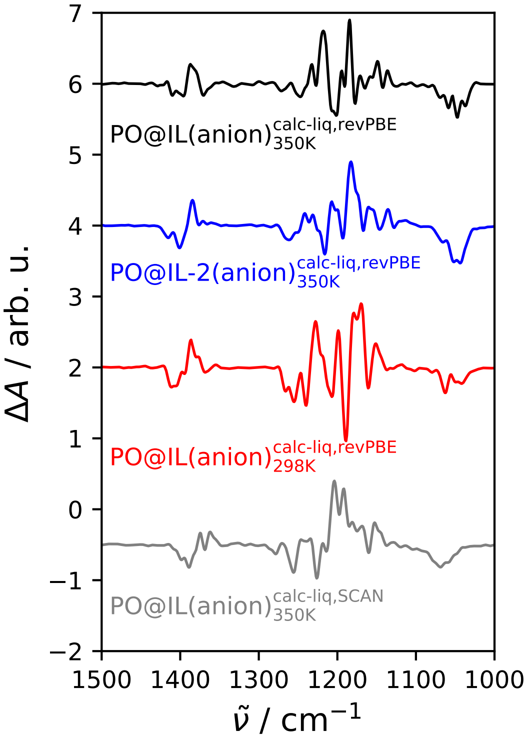

Figure S15: Theoretical global VCD spectra obtained for the systems PO@IL$^{revPBE}_{350K}$, PO@IL-2$^{revPBE}_{350K}$, PO@IL$^{revPBE}_{298K}$ and PO@IL$^{SCAN}_{350K}$. The frequencies of the VCD spectrum obtained for the PO@IL$^{SCAN}_{350K}$ system were scaled by a factor of 1.04.

References

(S1) Buffeteau, T.; Lagugné-Labarthet, F.; Sourisseau, C. Vibrational Circular Dichroism in General Anisotropic Thin Solid Films: Measurement and Theoretical Approach. *Appl. Spectrosc.* **2005**, *59*, 732–745.

(S2) Rey, I.; Johansson, P.; Lindgren, J.; Lassègues, J. C.; Grondin, J.; Servant, L. Spectroscopic and Theoretical Study of (CF3SO2)2N- (TFSI-) and (CF3SO2)2NH (HTFSI). *J. Phys. Chem. A* **1998**, *102*, 3249–3258.

(S3) Sobota, M.; Nikiforidis, I.; Hieringer, W.; Paape, N.; Happel, M.; Steinrück, H.-P.; Görling, A.; Wasserscheid, P.; Laurin, M.; Libuda, J. Toward Ionic-Liquid-Based Model Catalysis: Growth, Orientation, Conformation, and Interaction Mechanism of

the [Tf2N]- Anion in [BMIM][Tf2N] Thin Films on a Well-Ordered Alumina Surface. *Langmuir* **2010**, *26*, 7199–7207.

(S4) Heimer, N. E.; Del Sesto, R. E.; Meng, Z.; Wilkes, J. S.; Carper, W. R. Vibrational Spectra of Imidazolium Tetrafluoroborate Ionic Liquids. *J. Mol. Liq.* **2006**, *124*, 84–95.

(S5) Wan, S.; Sinclair, R. C.; Coveney, P. V. Uncertainty Quantification in Classical Molecular Dynamics. *Philos. Trans. Royal Soc. A* **2021**, *379*, 20200082.

(S6) Grossfield, A.; Patrone, P. N.; Roe, D. R.; Schultz, A. J.; Siderius, D.; Zuckerman, D. M. Best Practices for Quantification of Uncertainty and Sampling Quality in Molecular Simulations [Article v1.0]. *Liv. J. Comput. Mol. Sci.* **2019**, *1*, 5067–5067.

(S7) Soares, T. A.; Cournia, Z.; Naidoo, K.; Amaro, R.; Wahab, H.; Merz, K. M. J. Guidelines for Reporting Molecular Dynamics Simulations in JCIM Publications. *J. Chem. Inf. Model.* **2023**, *63*, 3227–3229.

(S8) Gardas, R. L.; Freire, M. G.; Carvalho, P. J.; Marrucho, I. M.; Fonseca, I. M. A.; Ferreira, A. G. M.; Coutinho, J. A. P. PρT Measurements of Imidazolium-Based Ionic Liquids. *J. Chem. Eng. Data* **2007**, *52*, 1881–1888.

(S9) Haynes, W. M., Ed. *CRC Handbook of Chemistry and Physics*, 97th ed.; CRC Press: Boca Raton, 2016.

(S10) Blasius, J.; Kirchner, B. Selective Chirality Transfer to the Bis(Trifluoromethylsulfonyl)Imide Anion of an Ionic Liquid. *Chem. Eur. J.* **2023**, e202301239.

(S11) Balasubramani, S. G.; Chen, G. P.; Coriani, S.; Diedenhofen, M.; Frank, M. S.; Franzke, Y. J.; Furche, F.; Grotjahn, R.; Harding, M. E.; Hättig, C.; Hellweg, A.; Helmich-Paris, B.; Holzer, C.; Huniar, U.; Kaupp, M.; Marefat Khah, A.; Karbalaei Khani, S.; Müller, T.; Mack, F.; Nguyen, B. D.; Parker, S. M.; Perlt, E.; Rap-

poport, D.; Reiter, K.; Roy, S.; Rückert, M.; Schmitz, G.; Sierka, M.; Tapavicza, E.; Tew, D. P.; van Wüllen, C.; Voora, V. K.; Weigend, F.; Wodyński, A.; Yu, J. M. TURBOMOLE: Modular Program Suite for Ab Initio Quantum-Chemical and Condensed-Matter Simulations. *J. Chem. Phys.* **2020**, *152*, 184107.

(S12) Ahlrichs, R.; Bär, M.; Häser, M.; Horn, H.; Kölmel, C. Electronic Structure Calculations on Workstation Computers: The Program System Turbomole. *Chem. Phys. Lett.* **1989**, *162*, 165–169.

(S13) Reiter, K.; Kühn, M.; Weigend, F. Vibrational Circular Dichroism Spectra for Large Molecules and Molecules with Heavy Elements. *J. Chem. Phys.* **2017**, *146*, 054102.

(S14) Perdew, J. P.; Burke, K.; Ernzerhof, M. Generalized Gradient Approximation Made Simple. *Phys. Chem. Lett.* **1996**, *77*, 3865–3868.

(S15) Perdew, J. P.; Burke, K.; Ernzerhof, M. Generalized Gradient Approximation Made Simple [Phys. Rev. Lett. 77, 3865 (1996)]. *Phys. Chem. Lett.* **1997**, *78*, 1396–1396.

(S16) Weigend, F.; Ahlrichs, R. Balanced Basis Sets of Split Valence, Triple Zeta Valence and Quadruple Zeta Valence Quality for H to Rn: Design and Assessment of Accuracy. *Phys. Chem. Chem. Phys.* **2005**, *7*, 3297.

(S17) Grimme, S.; Antony, J.; Ehrlich, S.; Krieg, H. A Consistent and Accurateab Initioparametrization of Density Functional Dispersion Correction (DFT-D) for the 94 Elements H-Pu. *J. Chem. Phys.* **2010**, *132*.

(S18) Grimme, S.; Ehrlich, S.; Goerigk, L. Effect of the Damping Function in Dispersion Corrected Density Functional Theory. *J. Chem. Phys.* **2011**, *32*, 1456–1465.

(S19) Bouř, P.; McCann, J.; Wieser, H. Measurement and Calculation of Absolute Rotational Strengths for Camphor, α-Pinene, and Borneol. *J. Phys. Chem. A* **1998**, *102*, 102–110.

(S20) Kühne, T. D.; Iannuzzi, M.; Del Ben, M.; Rybkin, V. V.; Seewald, P.; Stein, F.; Laino, T.; Khaliullin, R. Z.; Schütt, O.; Schiffmann, F.; others CP2K: An Electronic Structure and Molecular Dynamics Software Package-Quickstep: Efficient and Accurate Electronic Structure Calculations. *J. Chem. Phys.* **2020**, *152*, 194103.

(S21) VandeVondele, J.; Krack, M.; Mohamed, F.; Parrinello, M.; Chassaing, T.; Hutter, J. Quickstep: Fast and Accurate Density Functional Calculations Using a Mixed Gaussian and Plane Waves Approach. *Comput. Phys. Comm.* **2005**, *167*, 103–128.

(S22) Martínez, L.; Andrade, R.; Birgin, E. G.; Martínez, J. M. PACKMOL: A Package for Building Initial Configurations for Molecular Dynamics Simulations. *J. Comput. Chem.* **2009**, *30*, 2157–2164.

(S23) Plimpton, S. Fast Parallel Algorithms for Short-Range Molecular Dynamics. *J. Comput. Phys.* **1995**, *117*, 1–19.

(S24) Zhang, Y.; Yang, W. Comment on “Generalized Gradient Approximation Made Simple”. *Phys. Rev. Lett.* **1998**, *80*, 890–890.

(S25) Goedecker, S.; Teter, M.; Hutter, J. Separable Dual-Space Gaussian Pseudopotentials. *Phys. Rev. B* **1996**, *54*, 1703–1710.

(S26) Hartwigsen, C.; Goedecker, S.; Hutter, J. Relativistic Separable Dual-Space Gaussian Pseudopotentials from H to Rn. *Phys. Rev. B* **1998**, *58*, 3641–3662.

(S27) Krack, M. Pseudopotentials for H to Kr Optimized for Gradient-Corrected Exchange-Correlation Functionals. *Theor. Chem. Acc.* **2005**, *114*, 145–152.

(S28) VandeVondele, J.; Hutter, J. Gaussian Basis Sets for Accurate Calculations on Molecular Systems in Gas and Condensed Phases. *J. Chem. Phys.* **2007**, *127*, 114105.

(S29) Thomas, M.; Brehm, M.; Kirchner, B. Voronoi Dipole Moments for the Simulation of Bulk Phase Vibrational Spectra. *Phys. Chem. Chem. Phys.* **2015**, *17*, 3207–3213.

(S30) Thomas, M.; Kirchner, B. Classical Magnetic Dipole Moments for the Simulation of Vibrational Circular Dichroism by Ab Initio Molecular Dynamics. *J. Phys. Chem. Lett.* **2016**, *7*, 509–513.

(S31) Sun, J.; Ruzsinszky, A.; Perdew, J. P. Strongly Constrained and Appropriately Normed Semilocal Density Functional. *Phys. Rev. Lett.* **2015**, *115*, 036402.

(S32) Pádua, A. A. H.; Canongia Lopes, J. N.; Deschamps, J. Modeling Ionic Liquids Using a Systematic All-Atom Force Field. *J. Phys. Chem. B* **2004**, *108*, 2038–2047.

(S33) Pádua, A. A. H.; Canongia Lopes, J. N.; Deschamps, J. Modeling Ionic Liquids Using a Systematic All-Atom Force Field. *J. Phys. Chem. B* **2004**, *108*, 11250–11250.

(S34) Pádua, A. A. H.; Canongia Lopes, J. N. Molecular Force Field for Ionic Liquids Composed of Triflate or Bistriflylimide Anions. *J. Phys. Chem. B* **2004**, *108*, 16893–16898.

(S35) Pádua, A. A. H.; Canongia Lopes, J. N. Molecular Force Field for Ionic Liquids III: Imidazolium, Pyridinium, and Phosphonium Cations; Chloride, Bromide, and Dicyanamide Anions. *J. Phys. Chem. B* **2006**, *110*, 19586–19592.

(S36) Pádua, A. A. H.; Canongia Lopes, J. N.; Shimizu, K. Molecular Force Field for Ionic Liquids IV: Trialkylimidazolium and Alkoxycarbonyl-Imidazolium Cations; Alkylsulfonate and Alkylsulfate Anions. *J. Phys. Chem. B* **2008**, *112*, 5039–5046.

(S37) Pádua, A. A. H.; Shimizu, K.; Almantariotis, D.; Gomes, M. F. C.; Canongia Lopes, J. N. Molecular Force Field for Ionic Liquids V: Hydroxyethylimidazolium, Dimethoxy-2- Methylimidazolium, and Fluoroalkylimidazolium Cations and Bis(Fluorosulfonyl)Amide, Perfluoroalkanesulfonylamide, and Fluoroalkylfluorophosphate Anions. *J. Phys. Chem. B* **2010**, *114*, 3592–3600.

(S38) Neumann, J.; Golub, B.; Odebrecht, L.-M.; Ludwig, R.; Paschek, D. Revisiting Imidazolium Based Ionic Liquids: Effect of the Conformation Bias of the [NTf2] Anion Studied by Molecular Dynamics Simulations. *J. Chem. Phys.* **2018**, *148*, 193828.

(S39) Goloviznina, K.; Canongia Lopes, J. N.; Costa Gomes, M.; Pádua, A. A. H. Transferable, Polarizable Force Field for Ionic Liquids. *J. Chem. Theory Comput.* **2019**, *15*, 5858–5871.

(S40) Goloviznina, K.; Gong, Z.; Costa Gomes, M. F.; Pádua, A. A. H. Extension of the CL&Pol Polarizable Force Field to Electrolytes, Protic Ionic Liquids, and Deep Eutectic Solvents. *J. Chem. Theory Comput.* **2021**, *17*, 1606–1617.

(S41) Jorgensen, W. L.; Maxwell, D. S.; Tirado-Rives, J. Development and Testing of the OPLS All-Atom Force Field on Conformational Energetics and Properties of Organic Liquids. *J. Am. Chem. Soc.* **1996**, *118*, 11225–11236.

(S42) Lamoureux, G.; Roux, B. Modeling Induced Polarization with Classical Drude Oscillators: Theory and Molecular Dynamics Simulation Algorithm. *J. Chem. Phys.* **2003**, *119*, 3025–3039.

(S43) Padua, A. Fftool. Zenodo, 2015.

(S44) Son, C. Y.; McDaniel, J. G.; Cui, Q.; Yethiraj, A. Proper Thermal Equilibration of Simulations with Drude Polarizable Models: Temperature-Grouped Dual-Nosé–Hoover Thermostat. *J. Phys. Chem. Lett.* **2019**, *10*, 7523–7530.

(S45) Evans, D. J.; Holian, B. L. The Nose–Hoover Thermostat. *J. Chem. Phys.* **1985**, *83*, 4069–4074.

(S46) Shinoda, W.; Shiga, M.; Mikami, M. Rapid Estimation of Elastic Constants by Molecular Dynamics Simulation under Constant Stress. *Phys. Rev. B* **2004**, *69*, 134103.

(S47) Neese, F. Software Update: The scpORCA/Scp Program System—Version 5.0. *Wiley Interdiscip. Rev.: Comput. Mol. Sci.* **2022**, e1606.

(S48) Grimme, S.; Hansen, A.; Ehlert, S.; Mewes, J.-M. r2SCAN-3c: A "Swiss Army Knife" Composite Electronic-Structure Method. *J. Chem. Phys.* **2021**, *154*, 064103.

(S49) Brehm, M.; Kirchner, B. TRAVIS - A Free Analyzer and Visualizer for Monte Carlo and Molecular Dynamics Trajectories. *J. Chem. Inf. Model.* **2011**, *51*, 2007–2023.

(S50) Brehm, M.; Thomas, M.; Gehrke, S.; Kirchner, B. TRAVIS—A Free Analyzer for Trajectories from Molecular Simulation. *J. Chem. Phys.* **2020**, *152*, 164105.

(S51) Thomas, M.; Brehm, M.; Fligg, R.; Vöhringer, P.; Kirchner, B. Computing Vibrational Spectra from Ab Initio Molecular Dynamics. *Phys. Chem. Chem. Phys.* **2013**, *15*, 6608–6622.

(S52) Schrödinger, LLC

(S53) Humphrey, W.; Dalke, A.; Schulten, K. VMD: Visual Molecular Dynamics. *J. Molec. Graphics* **1996**, *14*, 33–38.

(S54) Hunter, J. D. Matplotlib: A 2D Graphics Environment. *Comput. Sci. Eng.* **2007**, *9*, 90–95.

(S55) Canongia Lopes, J. N.; Shimizu, K.; Pádua, A. A. H.; Umebayashi, Y.; Fukuda, S.; Fujii, K.; Ishiguro, S.-i. A Tale of Two Ions: The Conformational Landscapes of Bis(Trifluoromethanesulfonyl)Amide and N,N-Dialkylpyrrolidinium. *J. Phys. Chem. B* **2008**, *112*, 1465–1472.

(S56) Becke, A. D. Density-Functional Thermochemistry. III. The Role of Exact Exchange. *J. Chem. Phys.* **1993**, *98*, 5648–5652.

(S57) Lee, C.; Yang, W.; Parr, R. G. Development of the Colle-Salvetti Correlation-Energy Formula into a Functional of the Electron Density. *Phys. Rev. B* **1988**, *37*, 785–789.

(S58) Grimme, S.; Ehrlich, S.; Goerigk, L. Effect of the Damping Function in Dispersion Corrected Density Functional Theory. *J. Comp. Chem.* **2011**, *32*, 1456–1465.

(S59) Weigend, F.; Ahlrichs, R. Balanced Basis Sets of Split Valence, Triple Zeta Valence and Quadruple Zeta Valence Quality for H to Rn: Design and Assessment of Accuracy. *Phys. Chem. Chem. Phys.* **2005**, *7*, 3297–3305.

(S60) Weigend, F. Accurate Coulomb-fitting Basis Sets for H to Rn. *Phys. Chem. Chem. Phys.* **2006**, *8*, 1057.

(S61) Philippi, F.; Pugh, D.; Rauber, D.; Welton, T.; A. Hunt, P. Conformational Design Concepts for Anions in Ionic Liquids. *Chem. Sci.* **2020**, *11*, 6405–6422.

(S62) Brehm, M.; Thomas, M. An Efficient Lossless Compression Algorithm for Trajectories of Atom Positions and Volumetric Data. *J. Chem. Inf. Model.* **2018**, *58*, 2092–2107.

(S63) Brehm, M.; Thomas, M. Optimized Atomic Partial Charges and Radii Defined by Radical Voronoi Tessellation of Bulk Phase Simulations. *Molecules* **2021**, *26*, 1875.
